# Novel genomic islands and a new *vanD*-subtype in the first sporadic VanD-type vancomycin resistant enterococci in Norway

**DOI:** 10.1371/journal.pone.0255187

**Published:** 2021-07-23

**Authors:** Mushtaq T. S. AL Rubaye, Jessin Janice, Jørgen Vildershøj Bjørnholt, Aleksandra Jakovljev, Maria Elisabeth Hultström, Arnfinn Sundsfjord, Kristin Hegstad

**Affiliations:** 1 Research Group for Host-Microbe Interactions, Department of Medical Biology, University of Tromsø (UiT) – The Arctic University of Norway, Tromsø, Norway; 2 Norwegian National Advisory Unit on Detection of Antimicrobial Resistance, Department of Microbiology and Infection Control, University Hospital of North-Norway, Tromsø, Norway; 3 Department of Clinical Microbiology, Oslo University Hospital, Oslo, Norway; 4 Institute of Clinical Medicine, University of Oslo, Oslo, Norway; 5 Department of Medical Microbiology, St Olavs Hospital, Trondheim, Norway; 6 Division of Nephrology, Department of Medicine, St Olavs Hospital, Trondheim, Norway; Hospital Universitario Ramon y Cajal, SPAIN

## Abstract

**Background:**

Vancomycin-resistant enterococci (VRE) represent several types of transferable vancomycin resistance gene clusters. The *vanD* type, associated with moderate to high level vancomycin resistance, has only sporadically been described in clinical isolates. The aim of this study was to perform a genetic characterization of the first VanD-type VRE strains detected in Norway.

**Methods:**

The VanD-type VRE-strains (n = 6) from two patient cases were examined by antimicrobial susceptibility testing and whole genome sequencing (WGS) to uncover Van-phenotype, strain phylogeny, the *vanD* gene clusters, and their genetic surroundings. The putative transferability of *vanD* was examined by circularization PCR and filter mating.

**Results:**

The VanD-type *Enterococcus faecium* (n = 4) and *Enterococcus casseliflavus* (n = 2) strains recovered from two cases (A and B), expressed moderate to high level vancomycin resistance (MIC 64—>256 mg/L) and various levels of teicoplanin susceptibility (MIC 2—>256 mg/L). WGS analyses revealed phylogenetically different *E*. *faecium* strains (A1, A2, and A3 of case A and B1 from case B) as well as *vanD* gene clusters located on different novel genomic islands (GIs). The *E*. *casseliflavus* strains (B2 and B3 of case B) were not clonally related, but harbored nearly identical novel GIs. The *vanD* cluster of case B strains represents a novel *vanD*-subtype. All the *vanD*-GIs were integrated at the same chromosomal site and contained genes consistent with a *Clostridiales* origin. Circular forms of the *vanD*-GIs were detected in all strains except B1. Transfer of *vanD* to an *E*. *faecium* recipient was unsuccessful.

**Conclusions:**

We describe the first VanD-type *E*. *casseliflavus* strains, a novel *vanD*-subtype, and three novel *vanD*-GIs with a genetic content consistent with a *Clostridiales* order origin. Despite temporal occurrence, case A and B *E*. *faecium* strains were phylogenetically diverse and harbored different *vanD* subtypes and *vanD*-GIs.

## Introduction

Vancomycin resistant enterococci (VRE) have become a global nosocomial problem three decades after the first description in the late 1980s [1]. Eight different acquired vancomycin resistance gene clusters (*vanA*, *vanB*, *vanD*, *vanG*, *vanE*, *vanL*, *vanM*, and *vanN*) have been identified [2]. The *vanC* gene cluster is intrinsic in *E*. *casseliflavus* and *E*. *gallinarum* [2]. In general, *van* gene clusters encode three groups of co-acting enzymes; 1) enzymes necessary for the synthesis of new peptidoglycan precursors, 2) enzymes that erase the inherent D-Ala-D-Ala-ending precursors, and 3) a two-component signal transduction system for inducible resistance [3]. The normal enterococcal cell wall side chain terminal residue D-Ala-D-Ala, to which vancomycin binds with high affinity, are replaced by D-Ala-D-Lac in *vanA*, *vanB*, *vanD*, and *vanM* gene clusters or D-Ala-D-Ser in the other *van* gene clusters [3]. Vancomycin binds to D-Ala-D-Ser with seven times lower affinity compared to D-Ala-D-Ala, causing low-level vancomycin resistance, while the binding affinity of vancomycin to D-Ala-D-Lac is almost 1000 times lower mediating high-level resistance [4]. The *vanA* and *vanB* clusters dominate worldwide, likely due to linkage to successful mobile genetic elements (MGEs) [5]. Although the *vanA*, *vanB*, and *vanD* clusters have a similar organization, the *vanD* gene clusters have so far only been sporadically described on chromosomal genomic islands (GIs) that have not been shown to be transferable between enterococci [6–9]. The *vanD* gene cluster has up till now been reported in five species of enterococci (*Enterococcus faecium*, *Enterococcus faecalis*, *Enterococcus gallinarum*, *Enterococcus avium*, and *Enterococcus raffinosus*) [10].

The VanD-phenotype is characterized by moderate to high level vancomycin resistance and various levels of susceptibility to teicoplanin [3, 11, 12]. The housekeeping *ddl* gene (D-Ala-D-Ala ligase) is often inactivated by mutations in *vanD* containing strains causing an impaired chromosomal peptidoglycan synthesis pathway and addiction to *vanD*-expression as the alternative peptidoglycan precursor pathway [3, 7, 13]. Based on sequence differences, there are five known subtypes of *vanD*. The sequence diversity in *vanD* gene cluster subtypes mostly is in the *vanY*_*D*_, *vanH*_*D*_, *vanD*, and *vanX*_*D*_ genes and at the intergenic sequence between the two operons of the cluster [11]. VanD VRE are rare and have only been reported sporadically from the Netherlands, France, Canada, Japan, Sweden, Australia, the US, and Brazil during the last decades [7, 8, 10, 12–18].

In this study, we aim to determine the genetic relatedness between the first Norwegian VanD-type VRE strains, their Van-phenotype, and the putative MGEs harbouring the *vanD*-gene cluster.

## Material and methods

### Case descriptions

#### Case A

A middle-aged previously healthy female presented with acute hepatic failure. An urgent transplantation with an ABO-incompatible liver was performed. At week eight, a subphrenic abscess was diagnosed supported by the growth of *E*. *coli* and *E*. *faecium* and treated by local drainage. In week 16, a new subphrenic abscess was diagnosed and a *vanD E*. *faecium* in pure culture was isolated from the abscess drainage pigtail catheter. Screening for fecal VRE-carriage at week 20 after transplantation yielded *vanD E*. *faecium*. Several negative rectal VRE-screening samples were obtained during the subsequent 9 months, except for one *vanC E*. *casseliflavus* strain. Several screening samples were collected during linezolid treatment. Antibiotic treatment was successfully terminated almost a year after the transplantation.

#### Case B

An elderly female, undergoing hemodialysis for the last five years after kidney transplant failure, presented with recurrent urinary tract infections (UTIs), predominantly caused by *Klebsiella pneumoniae*, but occasionally by *E*. *faecium*. Due to relapsing *Clostridioides difficile* infections (CDIs), she had received oral vancomycin prophylaxis the last three years. The urine yielded *vanD E*. *faecium* in pure culture. Repeated fecal VRE-screening (follow-up 2 years) revealed the presence of *vanD E*. *casseliflavus*, but not *vanD E*. *faecium*. The *vanD E*. *faecium* UTI was successfully treated with linezolid, while the *C*. *difficile* prophylaxis was changed to metronidazole.

Relevant case characteristics are summarized in Table 1. Antibiotic treatment and microbiological findings for case A are presented in S1 Fig.

**Table 1:** Relevant case characteristics.

Case	Underlying condition	Indication antimicrobial treatment	Antimicrobial treatment	Time to isolation of *vanD E*. *faecium*	Infection focus	Rectal carriage^#^	Hospital
**A**	Acute liver Tx—otherwise healthy	Postoperative sub-phrenic abscesses	Broad spectrum beta-lactams, vancomycin, trimethoprim/ sulfamethoxazole (PJP prophylaxis)	19 weeks post liver tx	Subphrenic abcess	*vanD E*. *faecium*, *E*. *casseliflavus*	1 and 2
**B**	Tx kidney failure, hemodialysis, recurrent UTIs and CDIs	Recurrent CDI	Vancomycin p.o. (CDI prophylaxis)	3 years from start of vancomycin prophylaxis	Urinary tract infection	*vanD E*. *casseliflavus*	3

Tx: transplantation, UTI: Urinary tract infection, CDI: *C*. *difficile* infection, PJP: Pneumocystis jiroveci pneumonia, p.o.: postoperative.

^#^ Fecal screening with CHROMagar^™^ VRE.

### Ethical approval

Since this study contain only limited anonymized patient data, the study was approved by the Data Protection Officer at Oslo University Hospital and the Chief of Department of Microbiology at St Olavs Hospital. The written consents of the patients were obtained to use anonymized data from their patient journal in publication of this work.

### VRE strains and data collection

The first two cases of VanD-type VRE were identified in Norway in 2017. The Norwegian National Advisory Unit on Detection of Antimicrobial Resistance received the strains for further characterization (Table 2). Three VanD-positive *E*. *faecium* (VanD-type VRE*fm*) (A1, A2, and A3) strains were isolated from case A. The strains of case A were recovered from a subphrenic abscess (A1 and A2) and through rectal screening (A3). A month later, a VanD-type VRE*fm* (B1) strain was isolated from the urinary tract in a hemodialysis patient (case B). Further, two *vanD*-positive *E*. *casseliflavus* strains were recovered from case B by rectal screening, three weeks (B2) and two years (B3) later. Both patients had received vancomycin treatment before the isolation of the VanD-type VRE.

**Table 2:** Relevant strain characteristics.

Strain ID	Strain name	Species	MLST	VAN*	TEC	AMP	LIN	GEN	Ddl ligase changes compared to *E*. *faecium* E1	Source	Isolation day
**A1**	VRE1736	*E*. *faecium*	1486	64	4	>8	<1	>500	S185 changed to F185	Abcess drainage	Day 1
**A2**	VRE1737	*E*. *faecium*	1486	>128	4	>8	<1	>500	S185 changed to F185	Abcess drainage	Day 1
**A3**	KresVRE0001	*E*. *faecium*	117	64	2	>8	2	<32	S319 changed to G319 ^#^	Screening	Day 10
**B1**	KresVRE0002	*E*. *faecium*	203	>256	>256	>8	2	>500	Truncated protein of 110 aa ^#^	Urine	Day 42
**B2**	KresVRE0003	*E*. *casseliflavus*	-	>256	>256	1	2	<2		Rectal screening	Day 65
**B3**	KresVRE0012	*E*. *casseliflavus*	-	>256	>8	<0,25	2	<32		Rectal screening	Day 665

*, MICs in mg/L for VAN (vancomycin), TEC (teicoplanin), AMP (ampicillin), LIN (linezolid), and GEN (gentamicin).

^#^, These changes are not within the part of the *ddl* gene used for sequence typing.

### Antimicrobial Susceptibility Testing (AST) and *van* genotype determinations

AST was performed by broth microdilution using the GPALL1F or EUENCF Sensititre plates (Thermo Fisher Scientific, Waltham, Massachusetts, USA), ComASP^™^ Vancomycin, and Teicoplanin MIC Test Strip (Liofilchem, Roseto Degli Abruzzi, Italy). The results (MICs) were interpreted according to EUCAST clinical breakpoints v. 10.0 2020 [19]. The *van* genotype was initially determined by a *vanDEG* multiplex PCR as described previously [20, 21] and JumpStart REDTaq ReadyMix (Merck KGaA, Darmstadt, Germany). DNA extractions for PCRs were performed using the NucliSens EasyMAG instrument and reagents (BioMeriéux, Marcy-l’Étoile, France) according to the manufacturer’s instructions.

### Species identification and Whole Genome Sequencing (WGS)

Strains were subcultured on blood agar to ensure pure culture. Species identification was performed by MALDI-TOF (Bruker, Billerica, USA) according to the manufacturer’s instructions. Genomic DNA was extracted using DNeasy Blood and tissue kit (Qiagen, Hilden, Germany). The total DNA concentration was quantified by Qubit fluorometer (Invitrogen, Thermo Fisher Scientific). Libraries were prepared by the Nextera XT DNA library preparation kit (Illumina, San Diego, USA) and sequenced using Illumina NextSeq500 and the Mid Output 300 cycles cell.

### Genomic analyses

Adapter removal and quality trimming of the raw reads were performed by trimmomatic v0.39 [22]. Later, genome assembly was done using SPAdes v3.13.0 [23] and the quality of assembled genomes was assessed using QUAST v5.0.2 [24]. The annotation of the transposons was carried out using the National Center for Biotechnology Information (NCBI) prokaryotic genome annotation pipeline (PGAP) [25]. Antimicrobial resistance (AMR) genes were identified *in silico* from the assemblies using NCBI bacterial AMR reference gene database (PRJNA313047) [26] in ABRicate tool v0.8.7 [27]. Identification of Type IV secretion systems genes was carried out by BLASTp [28] searches against the SecReT4 database [29].

### Phylogenetic analyses

To explore the phylogenetic relationship between the *vanD* strains and publically available genome sequences on NCBI, the global phylogenetic trees were generated based on the core genome. All closed genomes of *E*. *faecium* (n = 135) and *E*. *casseliflavus* (n = 3) from NCBI as of 04.04.2020 were retrieved and phylogenetic trees were constructed using Parsnp v1.2 [30]. Another core genome SNP tree was built for the publicly available VanD-type VRE*fm* genome sequences together with the Norwegian vanD-type VRE*fm*. Also, a SNP tree was generated for *vanD* gene cluster sequences using parsnp. Multilocus Sequence Typing (MLST) was performed using MLST tool version 2.11 [31]. For high-resolution typing, Minimum Spanning Tree was generated based on the 1423 core genes of *E*. *faecium* scheme of SeqSphere+ software V6.0.2 (Ridom GmbH, Münster, Germany [http://www.ridom.de/seqsphere/]). We used the default ≤ 20 allelic differences as a threshold for cluster calculation and clonal relatedness [32].

### Comparative genomics

The closest non-VRE strains to each of the Norwegian VanD-type VRE were selected from the global phylogenetic tree. We used Mauve [33] to sort the contigs according to the reference genomes (E1 (NZ_CP018065.1) for A1-3 strains, E4402 (NZ_LR135174) for B1 strain, and EC20 (CP004856.1) for B2-3 strains) followed by Easyfig v2.2.2 [34] for comparison. The Artemis comparison tool [35] was used to visualize the BLASTn v2.6.0 search result and to locate the mobile genetic structures containing *vanD* gene clusters and their insertion site in the genome. Sequences of the GIs harboring the *vanD* gene clusters were BLASTed against the NCBI nr database to find the homologous sequences. Pyani v0.2.7 was used to determine the average nucleotide identity (ANI) between genomes, GIs and *vanD* gene clusters [36]. For the novel GIs, transposon numbers were registered at the Transposon Registry [37].

### Excision of putative GIs

The ability of the GIs to circularize was examined by PCR using the following pair of primers which directed outwards from the GIs ends: 5´-GCGTGAGAAGCTGACAACAA-3´ and 5´-GTTTCAGCCGCCAACTATTC-3´. Subsequent Sanger sequencing of PCR products using BigDye 3.1 technology (Applied Biosystems, CA, USA) was performed to confirm the expected sequence.

### Transferability of putative GIs

Transferability of *vanD* gene clusters was examined as described previously [38] using *E*. *faecium* BM4105-RF [39] as a recipient. To determine transfer frequency, colony forming units were counted on Brain heart infusion agar with rifampicin (30 mg/L) and fusidic acid (20 mg/L), and/or vancomycin (8 mg/L).

## Results and discussion

Most of the reported VanD-type VRE have been sporadic clinical isolates [7, 10, 12, 13, 15]. Despite an increasing prevalence of VRE in Norway since 2010, only *vanA* and *vanB* have been reported until now [40]. The detection of VanD-type VRE from two different patients within two months in 2017, therefore raised a concern of facing a VanD-type VRE outbreak in Norway, although no obvious epidemiological link between the patients was identified. Thus, the pheno- and genotype of the six VanD VRE strains were examined (Table 2). All three VRE from case A were *E*. *faecium*, while in case B, one *E*. *faecium* and two *E*. *casseliflavus* were isolated. To our knowledge, B2 and B3 are the first VanD-type vancomycin resistant *E*. *casseliflavus* strains reported.

### AST results

The AST-results are summarized in Table 2. Briefly, all strains expressed high-level vancomycin resistance (MIC ≥ 64 mg/L), various levels of susceptibility to teicoplanin (MIC 2 mg/L to >256 mg/L), and susceptibility to linezolid. All four *E*. *faecium* strains were ampicillin resistant and three also demonstrated high-level gentamicin resistance.

*In silico* analysis showed that all strains contained the *vanD* gene cluster integrated into their chromosome. The *E*. *casseliflavus* genomes (B2 and B3) also contained the intrinsic *vanC* gene cluster [2]. In the *E*. *faecium* strain B1, alignment of the housekeeping D-Ala-D-Ala ligase deduced from the *ddl* gene sequence showed a truncated protein of only 110 amino acids caused by a deletion resulting in a frameshift and a premature stop codon (Table 2 and S2 Fig). All the other VanD-type VRE*fm* strains showed point mutations in essential positions that presumably could lead to a non-functional Ddl ligase. In the literature, most VanD-type VRE strains described have had an impaired Ddl ligase and are thus dependent on the constitutively expressed *vanD* cluster to synthesise peptidoglycan [10].

### The VanD *E*. *faecium* strains from the two cases were not closely related

**Fig 1:**
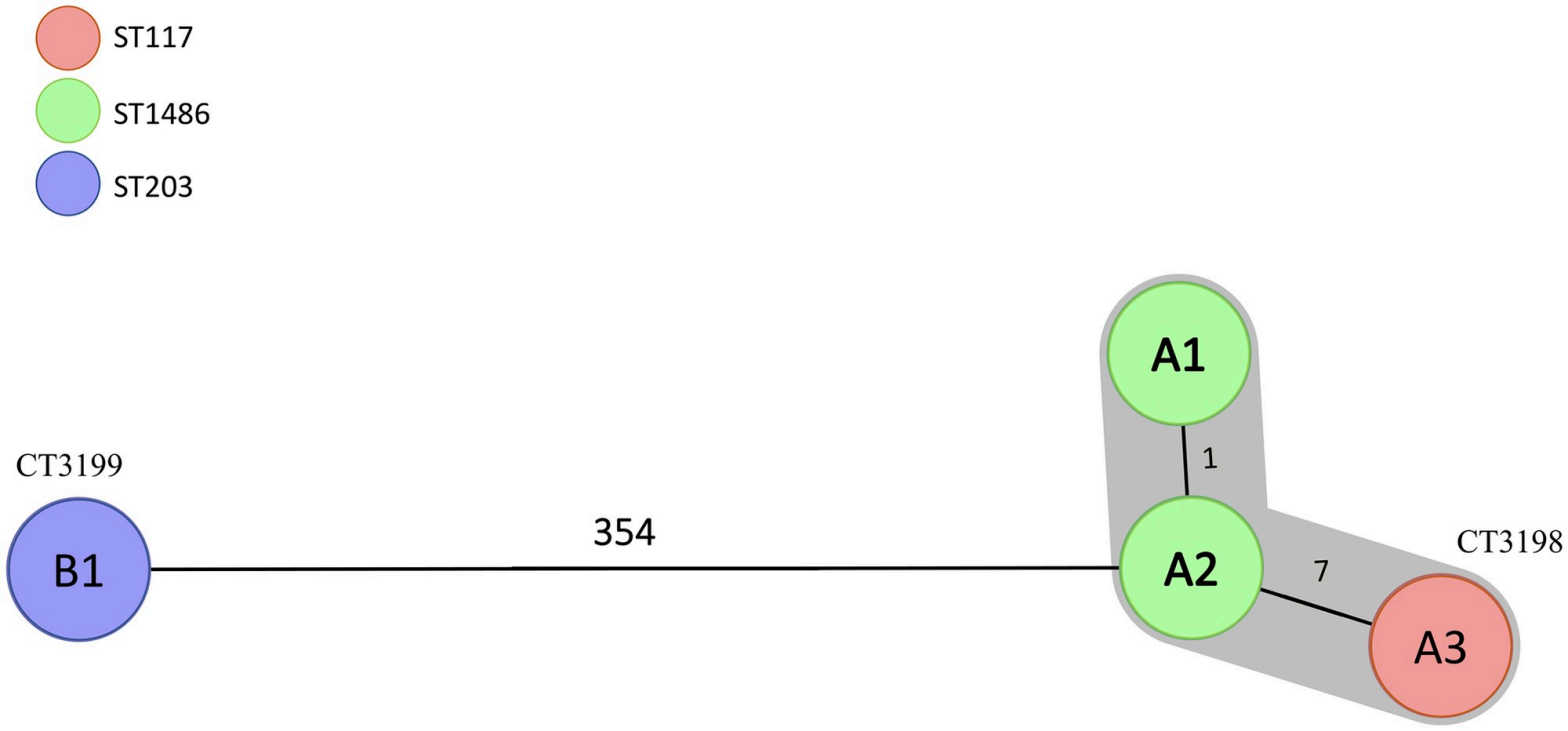
Minimum spanning tree based on the cgMLST typing of the Norwegian VanD VRE*fm*. Regardless of the different STs, A1-3 strains clustered together while the VRE*fm* strain B1 showed 354 allelic differences with the A2 strain.

The VanD VRE*fm* strains from cases A and B had different MLST profiles (Table 2). A1 and A2 genomes had an identical MLST profile which was registered as the novel ST1486, a single locus (*ddl* allele) variant of ST117 (strain A3) belonging to the hospital associated ST78 lineage. The *E*. *faecium* strain from case B belonged to ST203 which is part of the ST17 hospital associated lineage. Population genetic modeling based on the seven MLST genes using the Bayesian Analysis of Population Structure (BAPS) software have shown that 80% of the *E*. *faecium* nosocomial strains cluster in two different groups (2–1 and 3–3) [41]. *E*. *faecium* A and B strains belonged to lineages within these different main BAPS groups (lineage ST78 to 2–1 and lineage ST17 to 3–3) [41], confirming a large phylogenetic distance. This was further shown by cgMLST analysis which revealed that A1-3 strains belonged to the same novel cluster type (CT) 3198 (Fig 1). The B1 strain belonged to another novel CT3199 and showed at least 354 allelic differences to A1-3 strains. The two ST1486 strains had only one allelic difference, while the maximum allelic differences (eight) within CT3198 were between A1 and A3. One of these allelic differences was in the *ddl* allele which is one of the seven MLST scheme genes. Our results show that even strains with different MLST profiles could be clonally closely related and have the same CT.

For *E*. *casseliflavus* strains, a core genome SNP tree was constructed together with publically available closed genomes. Interestingly, the two VanD strains (B2 and B3) clustered in two separate branches, showing that they were not clonally related (S3 Fig).

The vancomycin susceptible *E*. *faecium* strain E1 (GCF_001886635.1) isolated from Spain in 2010, was identified as the closest genome to A1-3 strains using a core genome SNP tree of all closed *E*. *faecium* genomes in NCBI and the Norwegian VanD-type VRE*fm* genomes (S4 Fig). Strain E1 was therefore used as a reference genome for sorting contigs and further comparative genomic analyses. Genomic comparison using Easyfig confirmed that the A1-3 genomes were very similar. The ANI between A1 and A2 was the highest (99.99%). Comparison of case B VR*Efm* (B1) to case A VRE*fm* genomes, confirmed observed genomic differences (S5 Fig).

The significant phylogenetic difference between the *vanD E*. *faecium* strains from case A and B is consistent with the observed sporadic occurence of *vanD*-type VRE strains in contrast to the epidemic *vanA/B*-type VRE [7, 12, 13, 15]. Our patient characteristics with underlying diseases and long-term antibiotic exposure including vancomycin are also consistent with previous observations in *vanD* VRE cases [12, 17].

### A novel *vanD*-subtype was found in strains from case B

**Fig 2:**
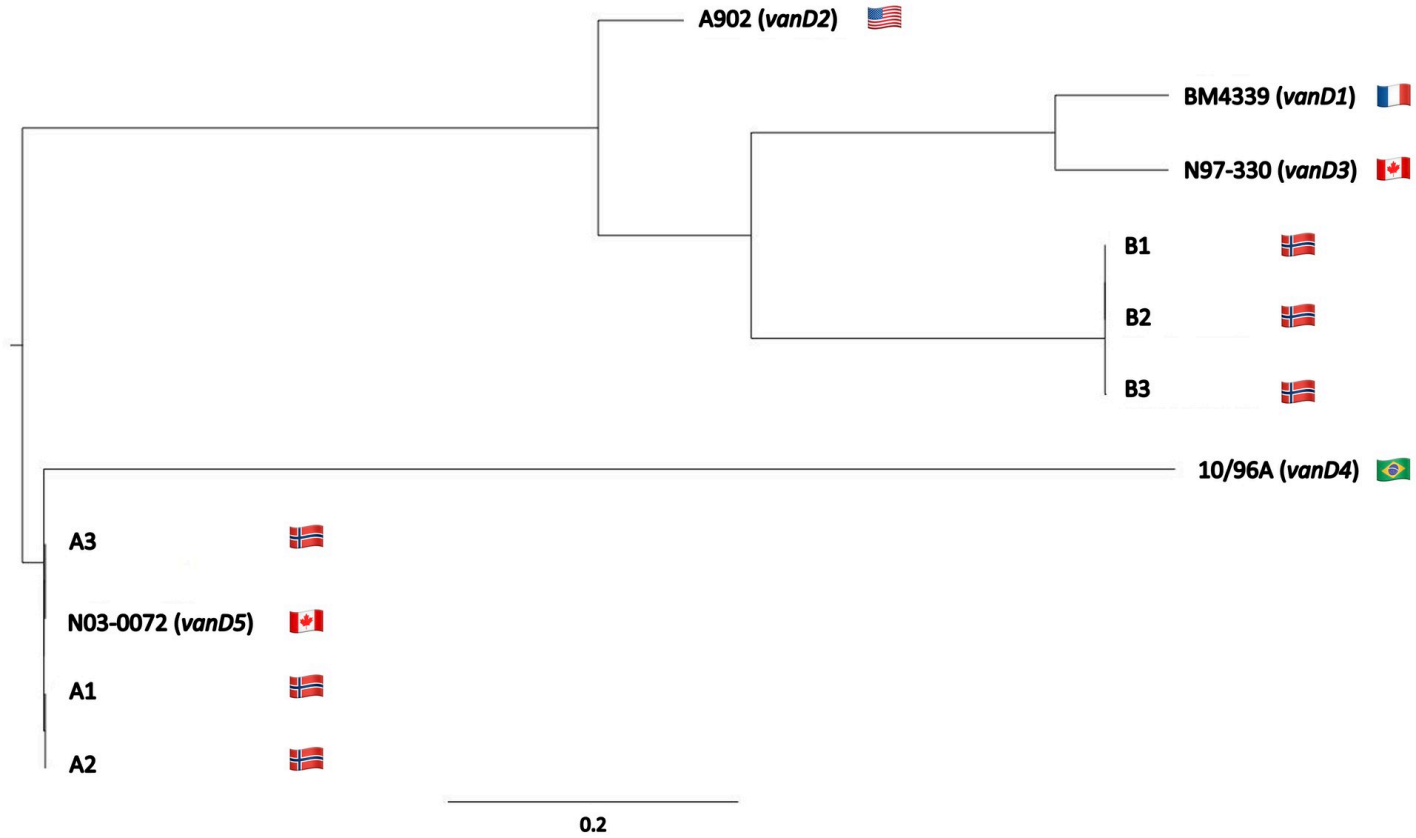
Phylogenetic SNP tree of the *vanD* gene clusters of the Norwegian and *vanD1*—*vanD5* subtype reference clusters retrieved from NCBI. Flags represent the countries that *vanD*-types were discovered in first. Case A strains clustered with *vanD5* reference N03-0072 while case B strains clustered separately.

Sequence comparison and phylogenetic analysis of complete *vanD* gene clusters from this study and reference sequences representing the five known *vanD* subtypes (*vanD1*-*D5*) [8, 11, 42, 43], showed that the Norwegian *vanD* gene clusters belonged to two different *vanD*-subtypes. In case A, the *vanD* gene clusters of strains A1 and A2 were 100% identical and showed 99.96% ANI to the cluster in A3. The *vanD* genes of case A clustered with the *vanD5* reference sequence (*E*. *faecium* strain N03-0072) (Fig 2). ANIs between the *vanD5* reference sequence and A1-3 strains were >99.9%. In case B strains, B2 and B3 *vanD* gene clusters were 99.98% identical and the B1 *vanD* gene cluster showed > 99.96% ANI with them. The ANI between case A and B *vanD* gene clusters was around 91%. B1-3 *vanD* gene clusters are significantly different from the known *vanD*-subtypes (maximum 93.7% identity to the known subtypes) (S1 Table). Thus, we propose that the B *vanD* gene cluster is a new subtype termed *vanD6*. Identification of the novel *vanD6* gene cluster in two different species of enterococci suggests interspecies genetic exchange.

### Three novel *vanD*-containing GIs identified

**Fig 3:**
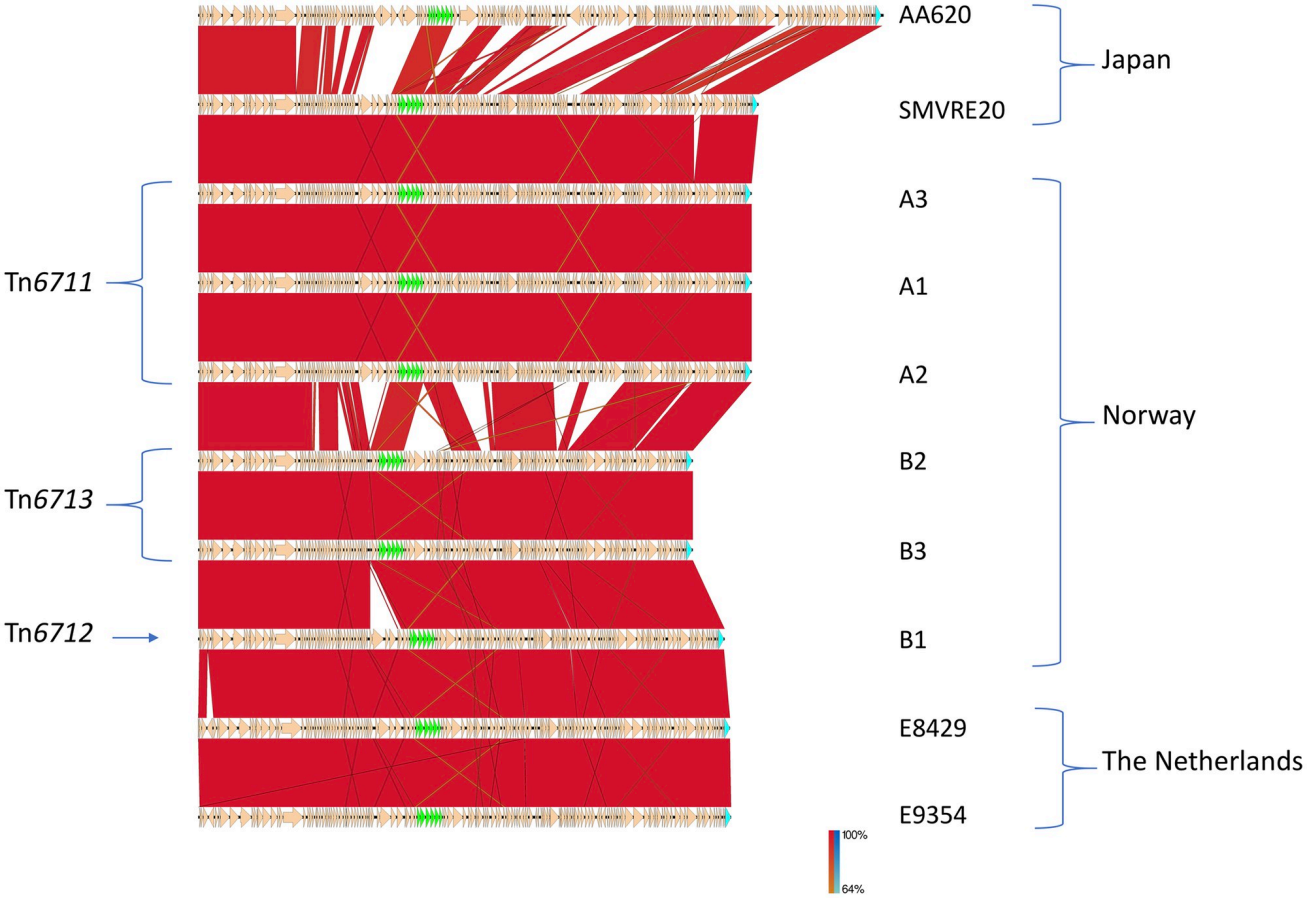
Comparison of the Norwegian, Dutch, and Japanese *vanD*-GIs built using Easyfig. A1-3 GIs have similar gene organization and showed high similarity with the Japanese SMVRE20 GI differing only in one hypothetical protein coding gene which contains transposase DDE domain. In case B, a high similarity exists between *E*. *casseliflavus* islands (B2 and B3) while the *E*. *faecium* island Tn*6712* of B1 is about 7.2 kb larger. The Dutch E8429 and E9354 showed the highest identity with case B GIs. *vanD* gene cluster and the integrase gene are marked in green and turquoise, respectively.

Comparison alignments with non-VRE reference genomes using Artemis comparison tool showed that all *vanD* gene clusters in the Norwegian vanD-type VRE were part of GIs ranging between 112–126 kb (Table 3). The GC content of the GIs was higher (44.1–44.3%) than the average GC content range of 38% of *E*. *faecium* strains [44–46]. For B2 and B3 *E*. *casseliflavus* strains, the genomic GC content was 42.4% and 42.3%, in contrast to 44.6 and 44.7% for their GIs, respectively. The GI Tn*6711* of A1-3 strains showed identical size and had an ANI above 99.99% suggesting a common origin. The GI Tn*6713* of the *E*. *casseliflavus* strains (B2 and B3) was identical in size and showed only 0.001% difference (S2 Table). The GI Tn*6712* in *E*. *faecium* strain B1 was 7230 bp larger than that of *E*. *casseliflavus* GI (Tn*6713*), while it was 6134 bp kb shorter and showed more rearrangements compared to Tn*6711* of strains A1-3 (Table 3 and Fig 3). ANIs were lowest (below 98%) between case A and B *E*. *faecium* GIs (S2 Table). Thus, the overall genetic differences between the GIs of A1-3 and B1-3, do not support a direct spread between the two cases. However, in case B strains, we suggest one genetic event has evolved Tn*6713* of *E*. *casseliflavus* to the longer Tn*6712* in *E*. *faecium* or vice versa (Fig 3).

**Table 3:** Characteristics of the GIs of the Norwegian VanD-type VRE.

Strain (case)	Genomic island				Repeats in the insertion site (5’-3’ strand)	
	Name	GC content (%)	Size in bp	Number of CDSs	*lysS* side	16S rRNA side*
A1 (A)	Tn*6711*	44.1	125858	157	TTCCCAACAATGA	TTCCCGACAATGA
A2 (A)	Tn*6711*	44.1	125858	157	TTCCCAACAATGA	TTCCCGACAATGA
A3 (A)	Tn*6711*	44.1	125858	157	TTCCCAACAATGA	TTCCCGACAATGA
B1 (B)	Tn*6712*	44.3	119724	149	TTCCCGACAATGA	TTCCCAACAATGA
B2 (B)	Tn*6713*	44.6	112494	143	TTCCCAACAATGA	TTCCCCACAATGA
B3 (B)	Tn*6713*	44.7	112494	143	TTCCCCACAATGA	TTCCCCACAATGA

*, difference compared to repeat on the *lysS* side is indicated by underlined nucleotide

All GIs lacked conjugative apparatus genes and the *vanD* gene cluster was the only AMR gene within the islands (GenBank Acc. No. MT951615-7). The nucleotide sequence of integrase genes in Tn*6712* and Tn*6713* was identical and had only one SNP compared to Tn*6711*. Despite the existence of the same GIs in *E*. *casseliflavus* strains (B1 and B2) of case B, the ANI between their genomes (95.1%) was too low to be clonally related. This observation strongly suggests separate acquisitions of Tn*6713* in B2 and B3 strains.

Comparisons of the Norwegian *vanD*-GIs to those of the newly isolated VanD-type VRE*fm* from the Netherlands and Japan with publically available WGS data revealed a high rate of identity. Two VanD-type Dutch VRE strains (E8429 and E9354) [7] contained *vanD*-GIs with 99.99% sequence identity to Tn*6712* of B1. Moreover, the *vanD5*-containing GI from the Japanese *E*. *faecium* SMVRE20 [17] (AP019408.1) showed 99.98% sequence identity to Tn*6711* of case A. Another Japanese *vanD*-GI (157 kb) from *E*. *faecium* strain AA620 (LC467712.1) showed 96% identity covering 81% of Tn*6711*. Although the *vanD*-GIs are similar between the Norwegian, Dutch, and Japanese VRE*fm* strains, phylogenetic analyses based on SNPs suggest that the strains are not closely related (S6 Fig). The GI of the Japanese SMVRE20 has an additional gene compared to Tn*6711*. Likewise, Tn*6712* and the Dutch GIs show only one gene in difference. Both these genes encode hypothetical proteins (Fig 3). The high identity between Tn*6711* and the GIs of the Japanese VanD-type VRE*fm* and between Tn*6712* and two Dutch VanD-type VRE*fm* GIs indicate a global spread of similar MGEs.

Due to the intrinsic *vanC* gene cluster of *E*. *casseliflavus* clinical strains, they already express low level resistance to vancomycin. Thus, *E*. *casseliflavus* strains often are not investigated further to see if they contain additional *van* clusters. In this study, we show that *E*. *casseliflavus* may be the intermediate source of the *vanD* type cluster containing GI (Tn*6713*) that spread to *E*. *faecium* (Tn*6712*) in case B. Based on this finding, MIC investigation of clinically important strains of *E*. *casseliflavus* should be considered to reveal possible acquired *van* gene clusters.

### The GIs show site specific integration in *E*. *faecium* and *E*. *casseliflavus*

The insertion sites of the *vanD* GIs were identical for all six strains and located in the 3’ end of the *lysS* gene which is positioned upstream of a 16S ribosomal rRNA gene. The integration resulted in a 13 bp direct repeat located 17 bp from the 3’ end of the *lysS* gene. The left and right repeats in the different *vanD*-containing strains showed maximum one SNP difference. For case A GIs the imperfect direct repeats were identical. In strain B1 of case B, the repeat is identical to case A GIs but localised on opposite sides. The perfect direct repeat in B3 differed by one nucleotide compared to the other strains (Table 3). The same integration site was also found in the recently isolated Dutch and Japanese VanD-type VRE*fm* [7, 17]. Thus, this insertion site may be a hotspot in some enterococcal species including *E*. *faecium* and *E*. *casseliflavus*.

### Putative origin of *vanD*-containing GIs

BLAST searches revealed 89% identity with several regions of *Blautia producta* SCSK genome covering only 59% of the Tn*6711* length. Another hit of Tn*6711* BLAST showed 89% identity to *Blautia coccoides* YL58 with 59% coverage, spanning some small fragments that were not covered by *B*. *producta* SCSK. An even higher identity (93%) was seen between the shorter Tn*6712* and Tn*6713* with fragments from *B*. *coccoides* YL58 covering 59% of these GIs. Previous reports have shown that *vanD*-type vancomycin resistance gene clusters can be found in non-enterococcal species like *Ruminococcus gauvreauii*, *Lachnospiraceae* bacterium, and *Ruthenibacterium lactatiformans* [7]. The above mentioned species and *Blautia* genus belong to the same taxonomic order of *Clostridiales* and are found in both the human and animal gut microbiome [47–49]. Thus, anaerobic *Blautia* genus or other members of the *Clostridiales* order are possible sources for *vanD* GIs.

### Activity and transferability of putative GIs

Mobile chromosomal genetic elements, excise and circularize before transfer [50]. Circularization PCR and amplicon sequencing confirmed that Tn*6711* and Tn*6713* were able to circularize supporting that they are active MGEs. Agarose gel electrophoresis of PCR products repeatedly showed stronger bands for Tn*6713* in *E*. *casseliflavus* which could be due to higher activity compared to Tn*6711* in *E*. *faecium* (S7 Fig). However, we were not able to transfer *vanD* to an *E*. *faecium* recipient in this study (detection limit 10^−10^ to 10^−9^ transconjugants/donor cell) which is not surprising since a conjugation apparatus was not found in any of the GIs carrying the *vanD* gene clusters nor in other sites of the VanD-type VRE genomes. Type IV secretion systems play an important role in conjugation and can mediate the transfer of the conjugative plasmids and transposons. They have an impact on the spread of antimicrobial resistance among bacteria [29]. Non-conjugative MGEs can use the conjugative apparatus of other MGEs to mobilize. Thus, a mobility test can be conducted to confirm mobilization of the GIs [38, 51]. However, the strains in this study already had several acquired resistance determinants that are used as markers in mobilization tests. Thus, we did not attempt to mobilize the islands.

## Conclusions

We have performed a genetic characterization of the first VanD-type VRE strains recovered from two patients treated with broadspectrum antibiotics including vancomycin before VRE detection. All VanD-type VRE strains of case A were *E*. *faecium* while both *vanD E*. *casseliflavus* and *E*. *faecium* were recovered from case B. To our knowledge, this is the first two *vanD E*. *casseliflavus* strains reported. Based on our finding, we recommend MIC investigation of clinically important *E*. *casseliflavus* strains to reveal possible additional *van* gene clusters. In the VRE*fm* strains of case A, we identified a unique novel ST1486, an SLV of ST117, which were phylogenetically distant from case B VRE*fm* (ST203). Sequence analyses revealed a novel *vanD*-type cluster termed *vanD6* subtype in case B strains. The large phylogenetic distance between the VRE*fm* strain of the two cases, as well as differences in *vanD*-cluster subtypes and *vanD*-GIs, rejected the hypothesis of a clonal outbreak. We identified three novel similar *vanD*-GIs of putative *Clostridiales* order origin integrated at the same chromosomal site in both *E*. *faecium* and *E casseliflavus*.

## Supporting information

S1 Fig Antibiotic treatment and microbiological findings for case A. Tx: Transplantation, BAL: Bronchoalveolar lavage.
(TIF)

S2 Fig Amino acid sequences alignment of the products deduced from the *ddl* genes of the *vanD*-containing *E*. *faecium* strains using Clustal omega online tool compared to the reference sequence (E1). Cov and pid represent the coverage and percent identity. The *ddl* gene of B1 showed a stop codon which resulted in a 110 amino acid protein. A1 and A2 showed a point mutation in a position involved in binding of D-Ala1 (S185 changed to F185) of the D-Ala:D-Ala ligase while A3 showed a point mutation in a position involved in binding of ATP (S319 changed to G319) [Depardieu F, Foucault M, Bell J, Dubouix A, Guibert M, Lavigne J, et al. New combinations of mutations in VanD-type vancomycin-resistant *Enterococcus faecium*, *Enterococcus faecalis*, and *Enterococcus avium* strains. Antimicrob Agents Chemother. 2009;53(5):1952–63]. The point mutations are highlighted by red boxes.
(TIF)

S3 Fig Core genome SNP tree for the Norwegian *E*. *casseliflavus* strains and the available closed genomes of the species in the NCBI database on 04.04.2020. (TIF)

S4 Fig Extended core genome SNP tree for all *E*. *faecium* closed genomes retrieved from the NCBI database on 04.04.2020 and VRE*fm* of this study. The Norwegian samples are colored red and the closest genomes to them are in green.
(TIF)

S5 Fig Genomic comparison between all Norwegian VanD-type VRE*fm* strains and *E*. *faecium* E1 reference genome using Easyfig tool. The red and blue gradient bars represent persent sequence matches. Red shows the direct and blue the inverted sequence matches. Arrows show the coding sequences and their direction. *vanD* gene cluster is marked in green. The similarities between case A strains (A1, A2 and A3) and their differences with case B VRE*fm* (B1) is reflected in their machting patterns.
(TIF)

S6 Fig Parsnp tree for the Norwegian, Dutch and Japanese VanD-type VRE*fm* genomes. Case A strains and the Japanese SMVRE20 which have the most identical GIs clustered separately. Likewise the Dutch E8429 and E9354 and B1 strain of case B also clustered separately.
(TIF)

S7 Fig Agarose gel electrophoresis of PCR products using pairs of primers directed outwards from the GI ends to confirm the presence of the active form of the GIs (circular form). All but B1 contain the active form.
(TIF)

S1 Table Average nucleotide identity between *vanD* gene cluster references (*vanD1*–*vanD5*) and the novel *vanD6* gene clusters from case B strains. (DOCX)

S2 Table Average nucleotide identity between GIs of the Norwegian VanD-type VRE samples. (DOCX)
