# Supplementary figures and images for: Novel genomic islands and a new vanD-subtype in the first sporadic VanD-type vancomycin resistant enterococci in Norway
Source: PLoS One. 2021 Jul 23;16(7):e0255187. doi: 10.1371/journal.pone.0255187 (PMC8301612; doi:10.1371/journal.pone.0255187)

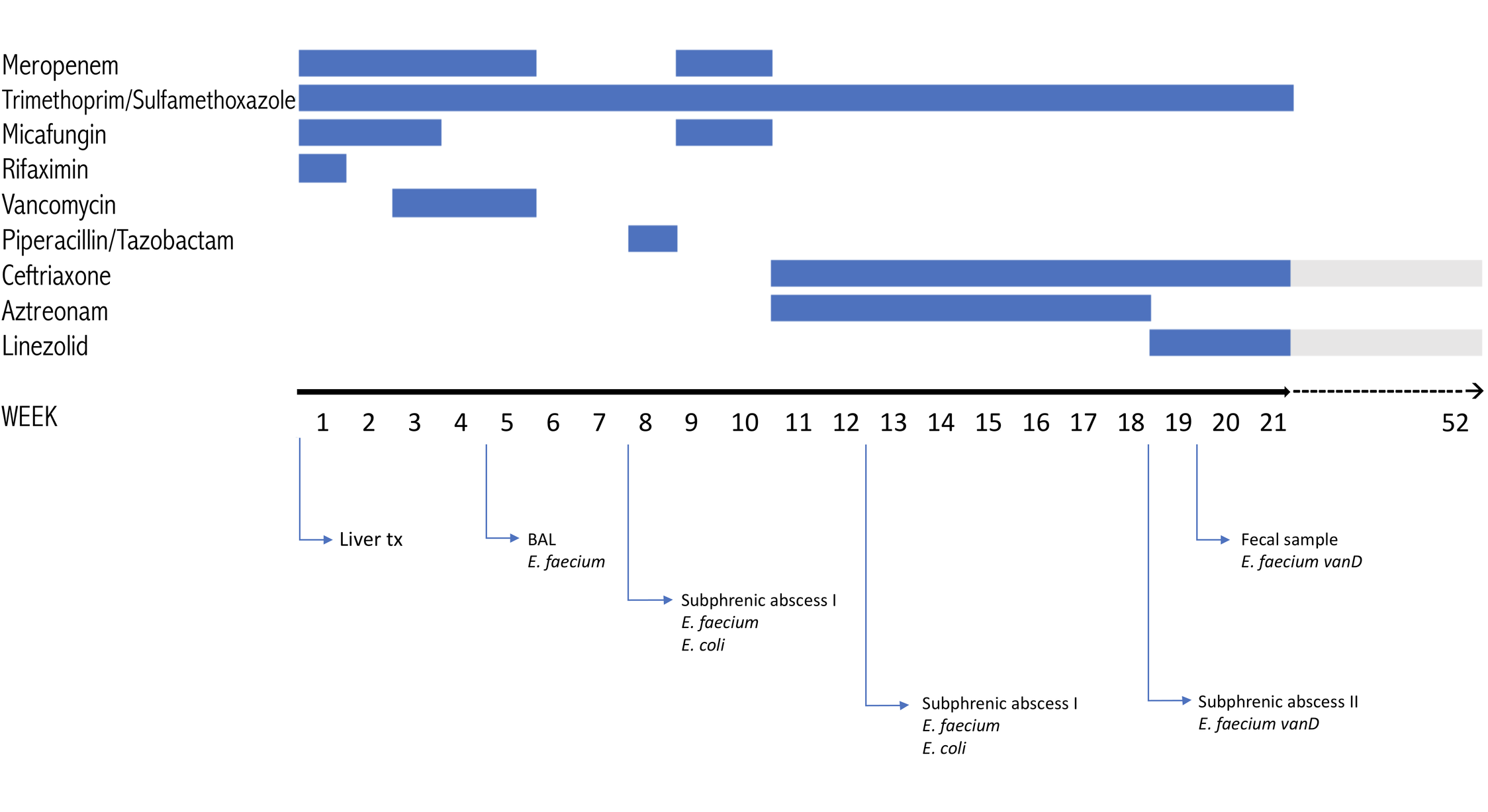

Supplement: S1 Fig — Tx: Transplantation, BAL: Bronchoalveolar lavage. (TIF) [file pone.0255187.s001.tif]

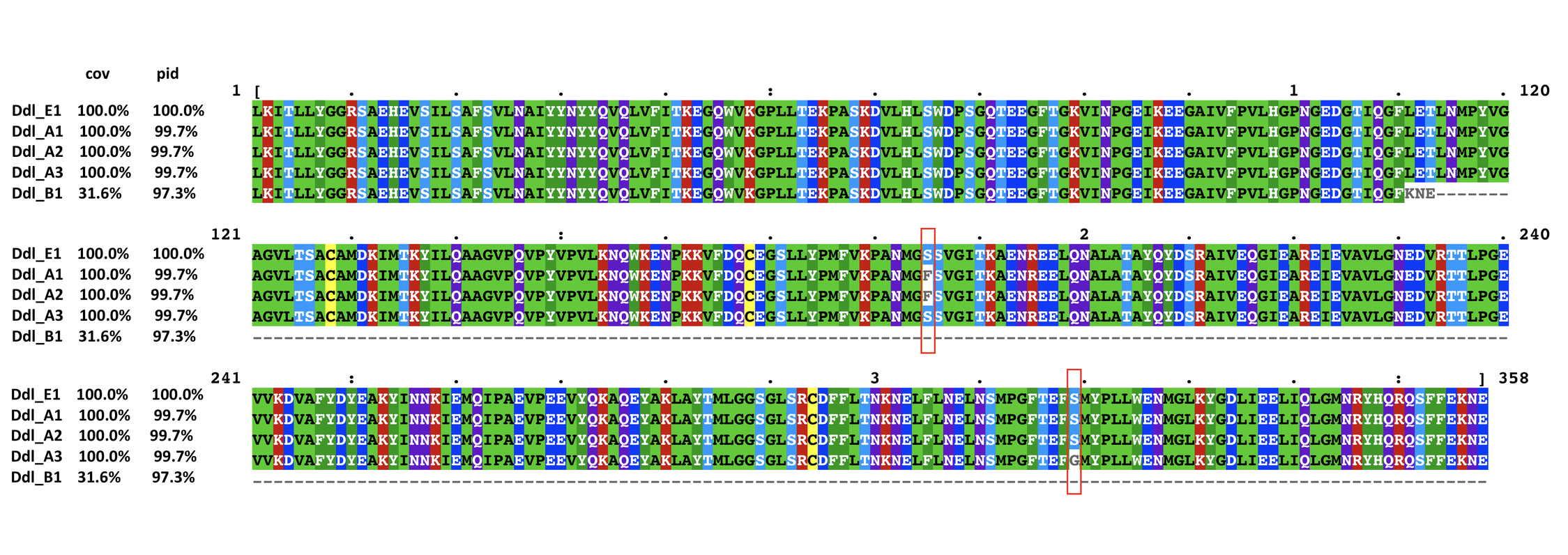

Supplement: S2 Fig — Cov and pid represent the coverage and percent identity. The ddl gene of B1 showed a stop codon which resulted in a 110 amino acid protein. A1 and A2 showed a point mutation in a position involved in binding of D-Ala1 (S185 changed to F185) of the D-Ala:D-Ala ligase while A3 showed a point mutation in a position involved in binding of ATP (S319 changed to G319) [Depardieu F, Foucault M, Bell J, Dubouix A, Guibert M, Lavigne J, et al. New combinations of mutations in VanD-type vancomycin-resistant Enterococcus faecium, Enterococcus faecalis, and Enterococcus avium strains. Antimicrob Agents Chemother. 2009;53(5):1952–63]. The point mutations are highlighted by red boxes. (TIF) [file pone.0255187.s002.tif]

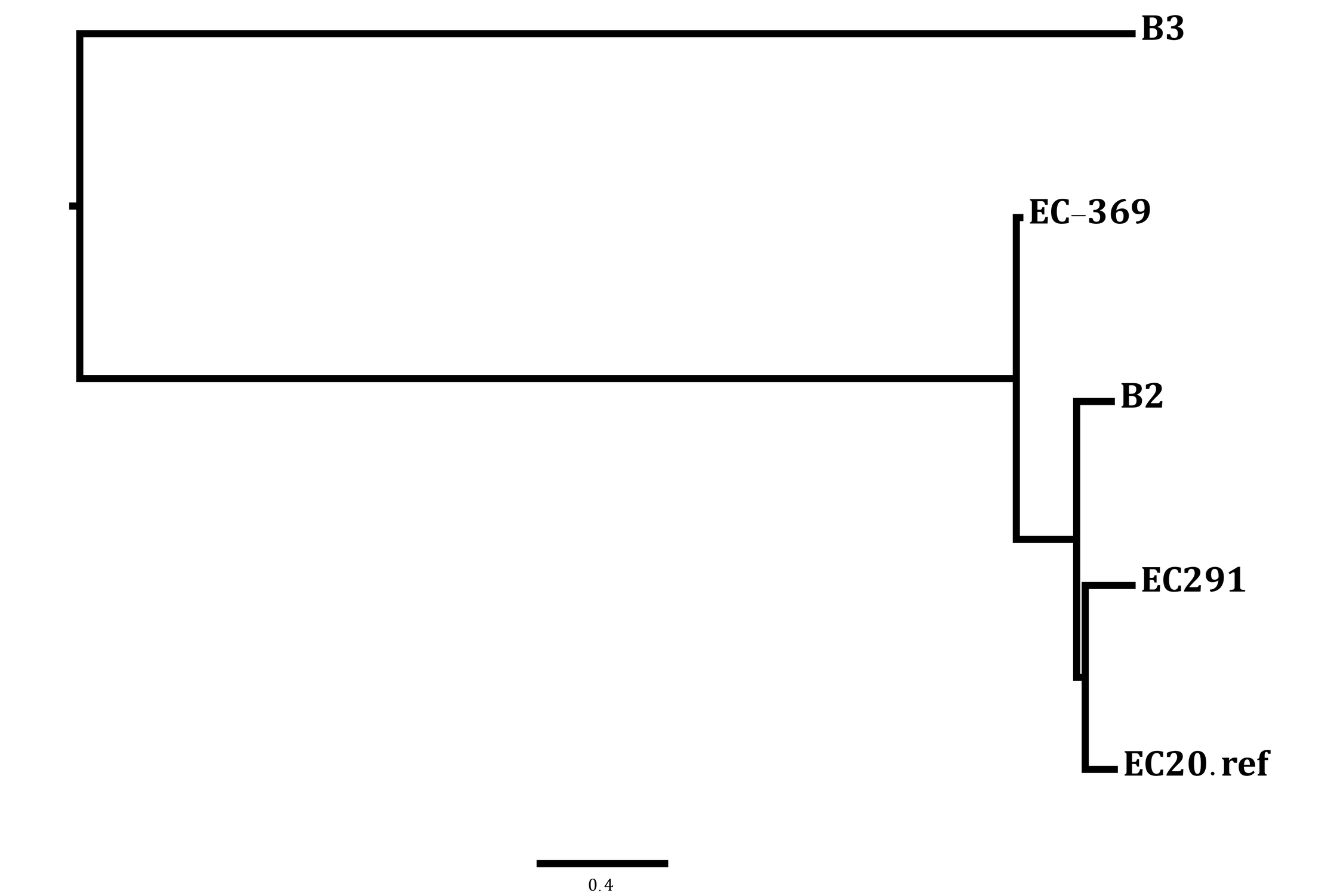

Supplement: S3 Fig — (TIF) [file pone.0255187.s003.tif]

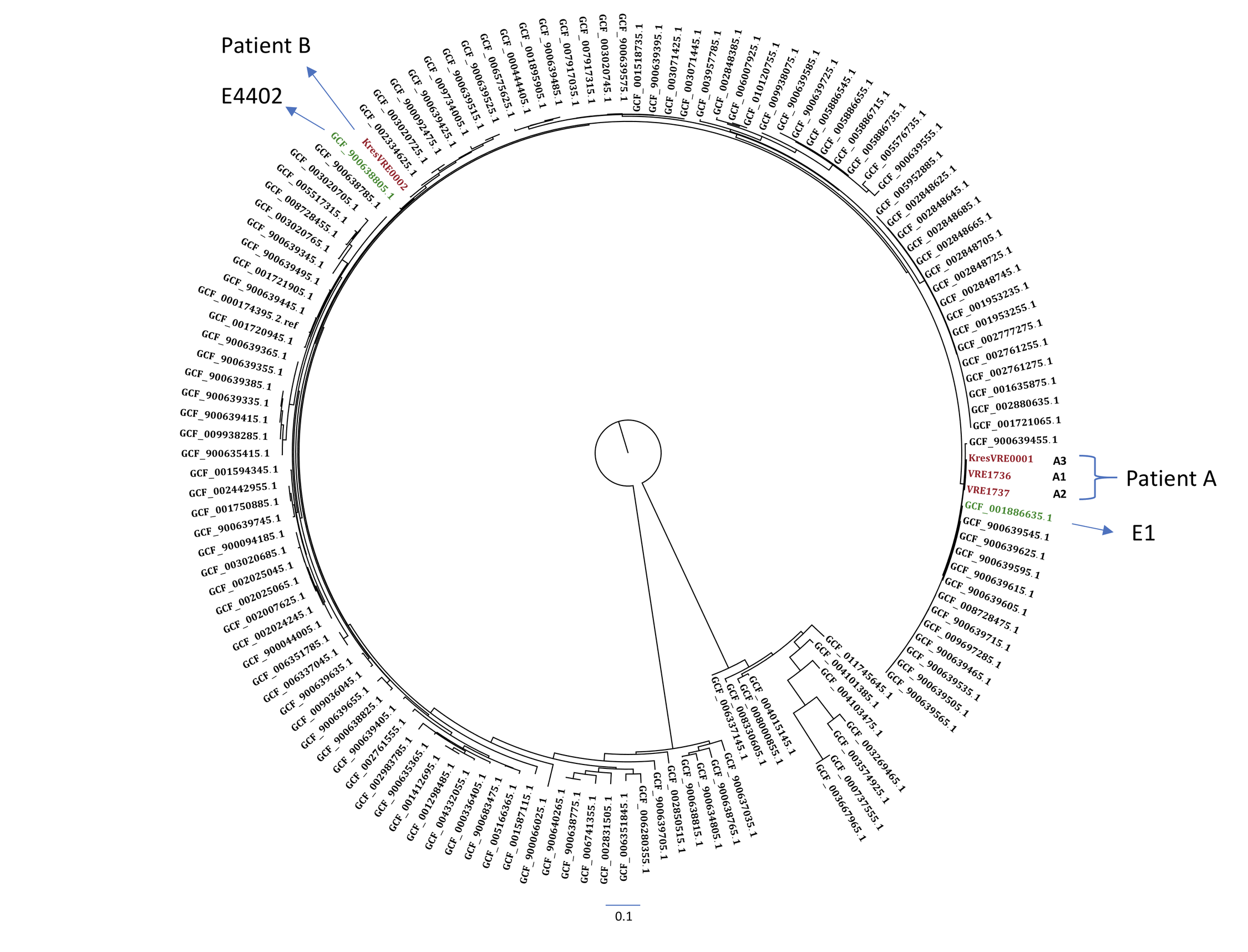

Supplement: S4 Fig — The Norwegian samples are colored red and the closest genomes to them are in green. (TIF) [file pone.0255187.s004.tif]

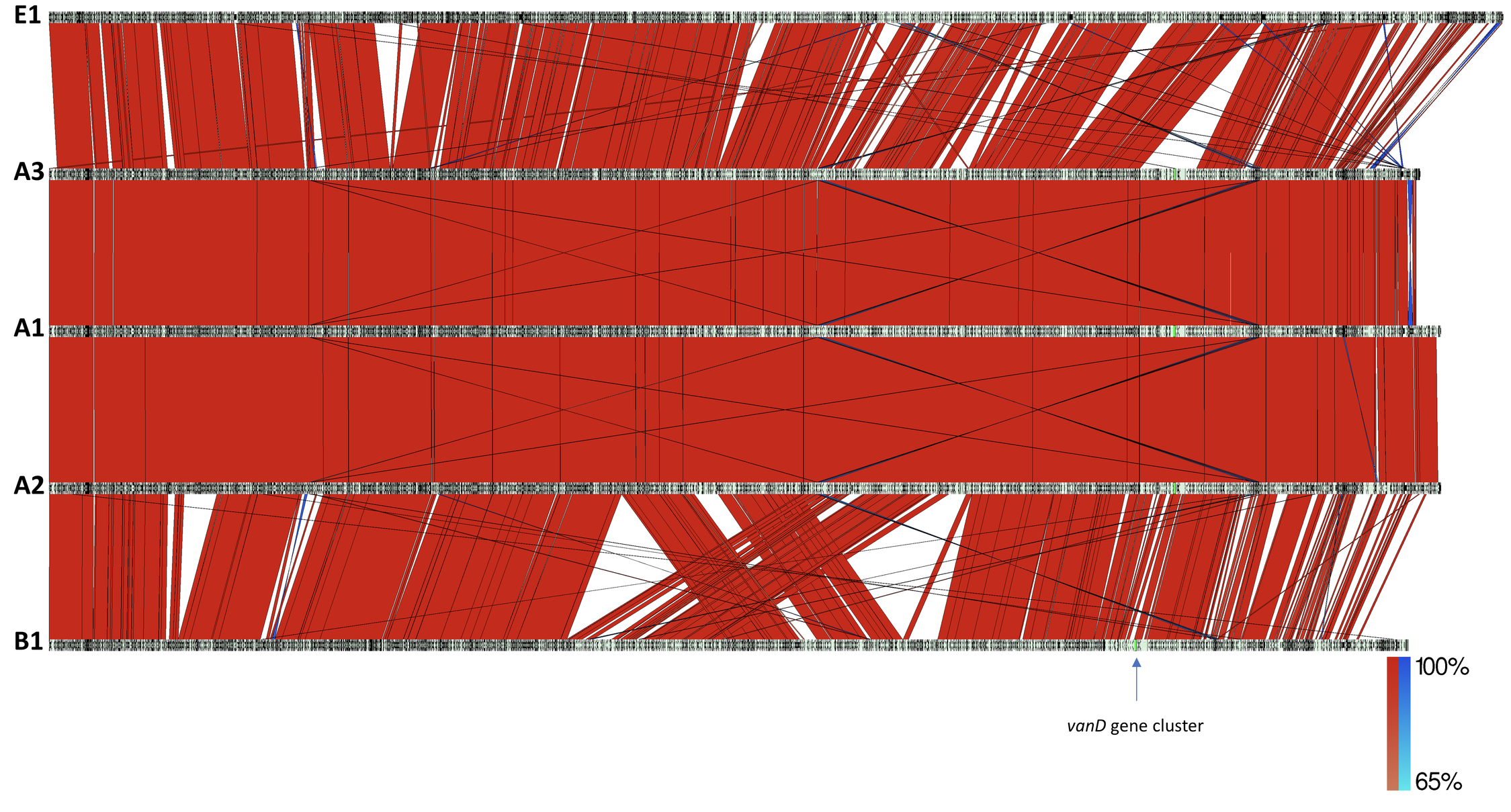

Supplement: S5 Fig — The red and blue gradient bars represent persent sequence matches. Red shows the direct and blue the inverted sequence matches. Arrows show the coding sequences and their direction. vanD gene cluster is marked in green. The similarities between case A strains (A1, A2 and A3) and their differences with case B VREfm (B1) is reflected in their machting patterns. (TIF) [file pone.0255187.s005.tif]

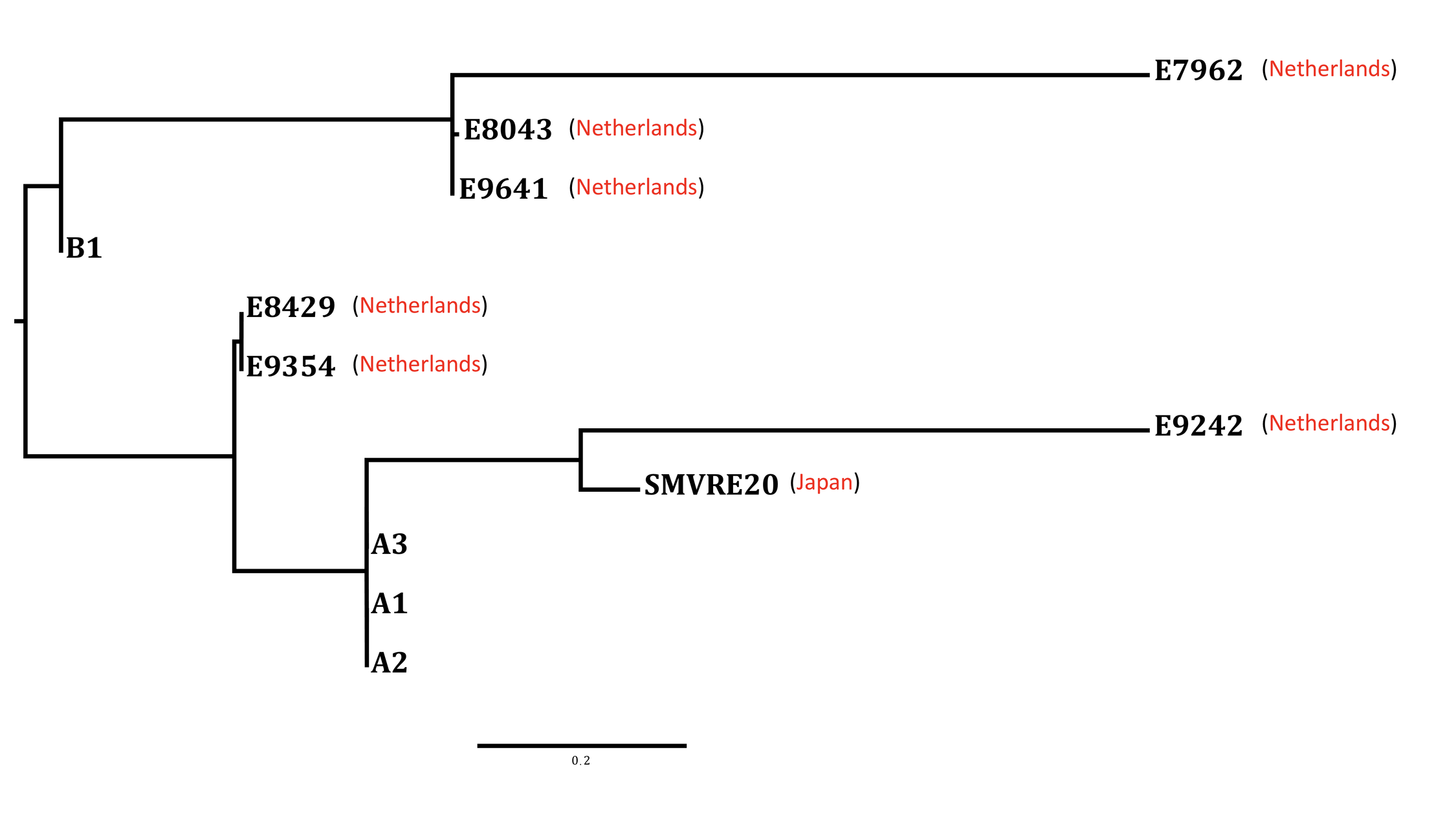

Supplement: S6 Fig — Case A strains and the Japanese SMVRE20 which have the most identical GIs clustered separately. Likewise the Dutch E8429 and E9354 and B1 strain of case B also clustered separately. (TIF) [file pone.0255187.s006.tif]

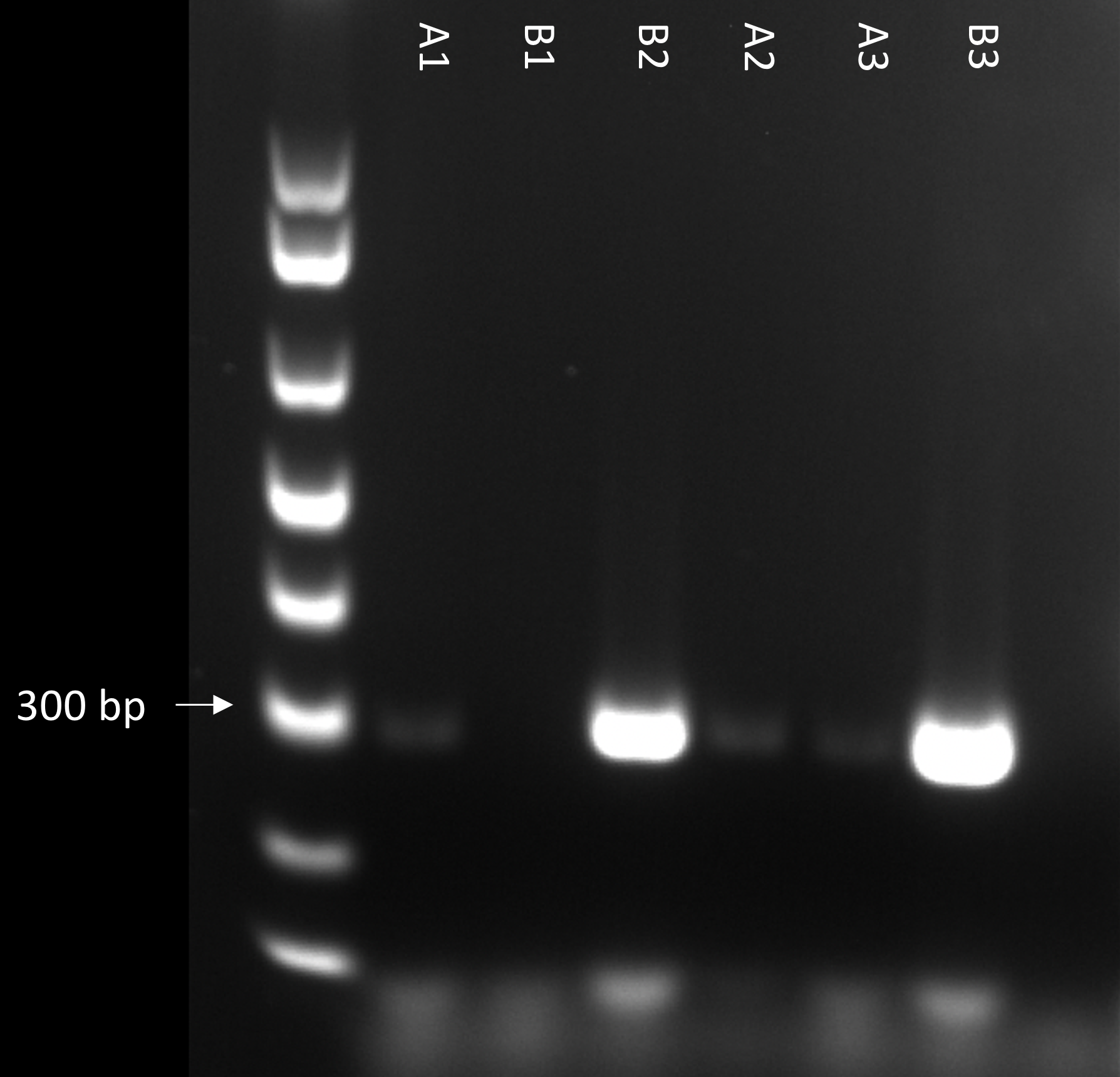

Supplement: S7 Fig — All but B1 contain the active form. (TIF) [file pone.0255187.s007.tif]
