# Supplementary material for: Novel genomic islands and a new vanD-subtype in the first sporadic VanD-type vancomycin resistant enterococci in Norway
Source: PLoS One. 2021 Jul 23;16(7):e0255187. doi: 10.1371/journal.pone.0255187 (PMC8301612; doi:10.1371/journal.pone.0255187)
Supplement: S1 Table — (DOCX) [file pone.0255187.s008.docx]

**S1 Table. Average nucleotide identity between *vanD* gene cluster references (*vanD1*–*vanD5*) and the novel *vanD6* gene clusters from patient B strains.**

|  | ***vanD1* Id (%)** | ***vanD2* Id (%)** | ***vanD3* Id (%)** | ***vanD4* Id (%)** | ***vanD5* Id (%)** |
| --- | --- | --- | --- | --- | --- |
| **B1 *vanD6*** | 92.300 | 93.725 | 92.633 | 87.700 | 91.595 |
| **B2 *vanD6*** | 92.317 | 93.742 | 92.650 | 87.721 | 91.612 |
| **B3 *vanD6*** | 92.300 | 93.726 | 92.633 | 87.700 | 91.595 |
