# Supplementary material for: Novel genomic islands and a new vanD-subtype in the first sporadic VanD-type vancomycin resistant enterococci in Norway
Source: PLoS One. 2021 Jul 23;16(7):e0255187. doi: 10.1371/journal.pone.0255187 (PMC8301612; doi:10.1371/journal.pone.0255187)
Supplement: S2 Table — (DOCX) [file pone.0255187.s009.docx]

**S2 Table. Average nucleotide identity between GIs of the Norwegian VanD-type VRE samples.**

|  | **A1 Tn*6711* Id (%)** | **A2 Tn*6711* Id (%)** | **A3 Tn*6711* Id (%)** | **B1 Tn*6712* Id (%)** | **B2 Tn*6713* Id (%)** | **B3 Tn*6713* Id (%)** |
| --- | --- | --- | --- | --- | --- | --- |
| **A1 Tn*6711*** | 100 | 99.999 | 99.997 | 97.823 | 97.680 | 97.678 |
| **A2 Tn*6711*** | 99.999 | 100 | 99.998 | 97.824 | 97.681 | 97.679 |
| **A3 Tn*6711*** | 99.997 | 99.998 | 100 | 97.824 | 97.682 | 97.681 |
| **B1 Tn*6712*** | 97.823 | 97.824 | 97.824 | 100 | 99.998 | 99.997 |
| **B2 Tn*6713*** | 97.680 | 97.681 | 97.682 | 99.998 | 100 | 99.999 |
| **B3 Tn*6713*** | 97.678 | 97.679 | 97.681 | 99.997 | 99.999 | 100 |
